# 185. Trends in Clinical Presentation and Antibiotic Resistance of Viridans Group Streptococci Bloodstream Infections in Immunocompromised Children

**DOI:** 10.1093/ofid/ofab466.185

**Published:** 2021-12-04

**Authors:** Ana M Quintero, Diego A Cruz Vidal, Monica I Ardura, Sophonie Jean

**Affiliations:** Nationwide Children’s Hospital, Columbus, OH

## Abstract

**Background:**

Levofloxacin prophylaxis (LVXp) is recommended in children with severe neutropenia from underlying malignancy or hematopoietic cell transplantation (HCT). The impact of LVXp on the epidemiology of viridans group streptococcus bloodstream infections (VGS-BSI) is unknown. At our center, LVXp was prescribed to high-risk children with expected prolonged neutropenia (ANC < 100, > 7 days) as part of a clinical trial (2013-17) and routinely since November 2018. We aim to describe our local epidemiology, antibiotic susceptibilities, and clinical outcomes of VGS-BSI over time.

**Methods:**

VGS-BSI from 1/1/10-1/31/21 were identified via the laboratory database. Clinical data of patients followed at NCH with underlying malignancy, severe neutropenia, or HCT were extracted from the electronic health record. Available VGS isolates were subcultured, species identification confirmed by MALDI-ToF or 16s rDNA sequencing and susceptibility to penicillin (PCN), cefepime (CEF), vancomycin (VAN), and LVX performed via Etest per CLSI M100 guidelines. Non-parametric descriptive statistics were applied.

**Results:**

Over a 10-yr period, 111 VGS-BSI occurred in 93 patients (Table 1); 15 (16%) patients had ≥ 2 VGS-BSI. 80 (86%) patients had fever and neutropenia (F&N); 26 (28%) required ICU care for vasopressors (N=17, 18%) or mechanical ventilation (N=10, 11%). Most VGS isolates were *S. mitis/oralis* group. In total, 15 (16%) patients received LVXp ≤ 6 months before VGS-BSI; 9 (10%) had breakthrough VGS-BSI while receiving LVXp and all isolates were LVX resistant. Figure 1 shows susceptibilities: overall, 24% of isolates had frank resistance to PCN, 19% CEF, 13% LVX; all were VAN susceptible. When evaluating for changes in susceptibilities over time, there was a significant difference in the proportion of LVX-resistant isolates (p=0.009, Cochran-Armitage χ ^2^), but not CEF (p=0.08) or PCN (p=0.86).

Table 1. Demographic and Clinical Characteristics of Immunocompromised Children with Viridans Group Streptococci Bloodstream Infections (VGS-BSI)

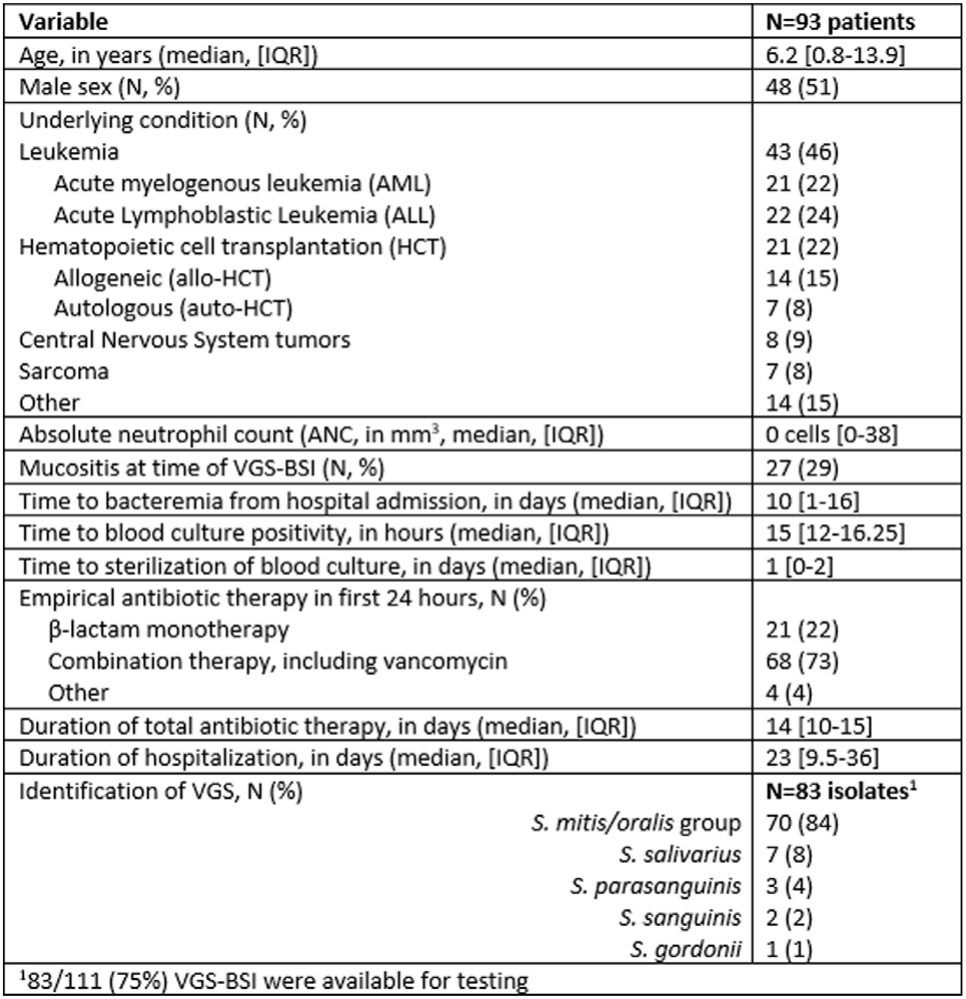

Figure 1. Antimicrobial Susceptibility Profile of Viridans Group Streptococci Bloodstream Isolates from Immunocompromised children, 2010-2021. Of 111 VGS-BSI reported during the study period from immunocompromised children, 83 (75%) were available for further testing. Antimicrobial susceptibility testing was performed by Etest and interpreted per CLSI M100. Susceptibility profiles to penicillin (PCN), cefepime (CEP) and, levofloxacin (LVX) are shown. Abbreviations: S—susceptible, I—intermediate, R—resistant.

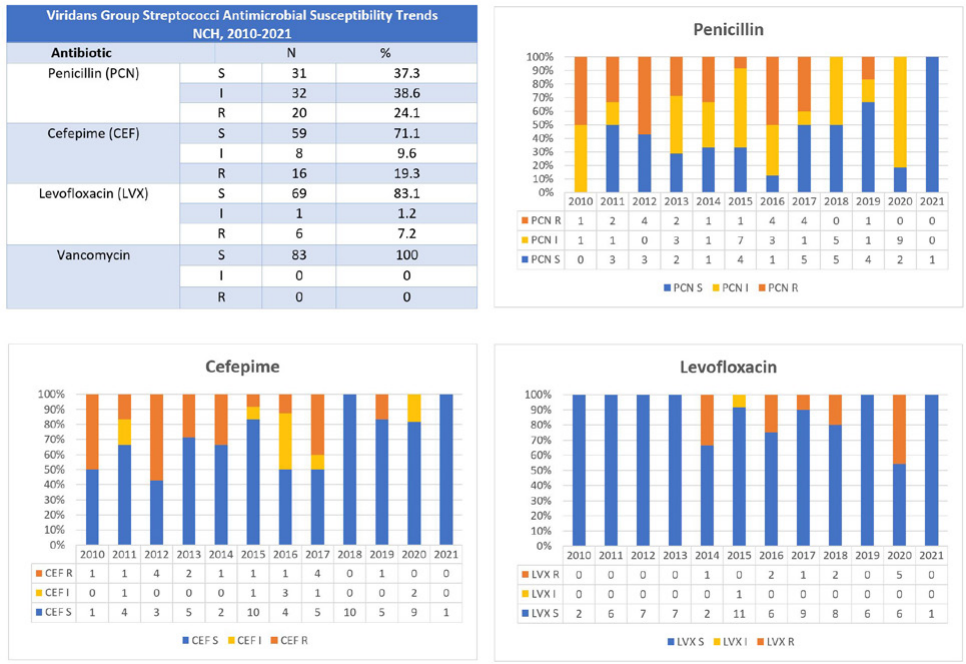

**Conclusion:**

Breakthrough, LVX-resistant VGS-BSI occurred in 10% of patients, most frequently in children with AML or HCT. Over time, there was a trend towards increased LVX resistance in the cohort. Routine antimicrobial testing and ongoing monitoring for emergence of resistance are warranted to inform local prophylaxis and empirical antibiotic strategies for high-risk children with F&N.

**Disclosures:**

**Monica I. Ardura, DO, MSCS**, **Shire** (Grant/Research Support)

